# External Oblique Intercostal Plane Block: A Case Report and Review of Literature

**DOI:** 10.7759/cureus.61617

**Published:** 2024-06-03

**Authors:** Praveen Reddy Elmati, Vikas Raghove, Gowthami Sai Kogilathota Jagirdhar, Alexander Bautista

**Affiliations:** 1 Department of Anesthesiology and Interventional Pain Medicine, Saint Clare’s Health, Denville, USA; 2 Department of Anesthesiology, IU (Indiana University) Health Ball Memorial Hospital, Muncie, USA; 3 Department of Internal Medicine, Saint Michael's Medical Center, Newark, USA; 4 Department of Anesthesiology, University of Louisville Hospital, Louisville, USA

**Keywords:** interventional pain medicine, ultrasound guidance, external oblique intercostal plane block, chest wall pain, spinal cord stimulation, post-laminectomy syndrome, chronic low back pain

## Abstract

Chronic low back pain (CLBP) and post-laminectomy syndrome (PLS) can pose significant therapeutic challenges, often refractory to conservative management. We present a case of a 52-year-old male with refractory CLBP and PLS who underwent spinal cord stimulation (SCS) lead placement, and subsequently developed chronic right anterior chest wall and upper abdominal pain. Despite using SCS and opioid therapy, the pain persisted until an ultrasound-guided external oblique intercostal plane block (EOIPB) was administered, resulting in complete pain relief. This case highlights the efficacy of EOIPB in managing chronic post-surgical neuropathic pain, underscoring its potential as a valuable intervention in such cases.

## Introduction

Fascial plane blocks in regional anesthesia have gained importance in recent years. It involves injection into a tissue plane to provide analgesia and is an alternative to neuraxial and paravertebral techniques. It is often safer and is associated with less cardiorespiratory instability or complications compared to epidural analgesia [1]. External oblique intercoastal plane block (EOIPB) is given at the mid-clavicular line and may block the anterior and lateral branches of intercostal nerves. Anesthesia is provided between the external oblique and intercostal nerves. This allows analgesia of the anterior upper abdominal wall between T6-T10 dermatomes in the anterior axillary line and T6-T9 dermatomes in the midline [2]. It blocks the lateral and anterior branches of T6-T10 intercostal nerves. Elsharkawy et al. report a case series of 22 patients receiving this block for upper abdominal wall analgesia [2]. It was used for bowel resection, liver transplant, exploratory laparotomy, nephrectomy, laparoscopic surgeries including Whipple’s, gastrectomy, proctopexy, and biliary surgeries. Most of the literature mentions using this regional analgesia in acute pain management. Informed consent was obtained before publication of this case report. We report a case of chronic low back pain (CLBP) who underwent spinal cord stimulation (SCS) and developed right anterior chest wall and upper abdominal pain after paddle lead placement. Despite chronic opioid therapy, the pain persisted until it was treated with an EOIPB under ultrasound guidance, resulting in complete pain relief. This case underscores the efficacy of fascial plane blocks in managing chronic post-surgical neuropathic pain.

## Case presentation

A 52-year-old male presented to our pain clinic with complaints of CLBP and bilateral leg pain that had been ongoing for over 10 years. The patient had a history of multiple lumbar spine surgeries for disc herniation and spinal stenosis, including laminectomies at the L4-L5 and L5-S1 levels. Despite these surgeries, the patient continued to experience severe, disabling pain that was refractory to conservative treatments, including physical therapy, epidural steroid injections, and opioid therapy. Given the failure of conservative management, the patient underwent percutaneous SCS lead placement at the L1-L2 level. Initially, the patient experienced significant pain relief; however, several months later, he began to notice a return of his lower back pain.

The patient subsequently underwent surgical revision of the SCS lead placement, with repositioning of the lead at the T8-T9 level. However, following this procedure, the patient developed chronic right chest wall pain that was described as sharp and stabbing in nature, exacerbated by movement and deep breathing. The pain was 6 to 7 on the numerical pain rating scale. The pain was refractory to opioid therapy and significantly impacted the patient's daily activities and quality of life. Imaging confirmed proper lead placement, ruling out mechanical causes for the pain.

Given the failure of conservative measures, including opioids, we opted for an interventional approach. We considered various interventional pain management techniques, including intercostal nerve blocks, paravertebral blocks, erector spinae blocks, peripheral nerve stimulation, and thoracic epidural steroid injections. After discussing with the patient and obtaining informed consent, we proceeded with an EOI block targeting the right anterior chest and upper abdomen wall involving the T7-T9 intercostal nerves. A mixture of triamcinolone (40 mg) and local anesthetics (10 mL of 0.25% bupivacaine) was injected, providing immediate and complete pain relief for eight months. The patient's patient improved from a 6/10 to 0/10 on the numerical pain rating scale.

## Discussion

Post-surgical neuropathic pain can significantly impact patient outcomes. In our case, right anterior chest wall pain following surgical paddle SCS lead placement posed a therapeutic challenge. Interventional techniques, such as ultrasound-guided EOIPB, offer an effective solution. EOIPB successfully covers both anterior and lateral cutaneous branches of intercostal nerves. EOIPB is commonly employed in acute pain management in open abdominal surgeries, particularly for alleviating discomfort in the chest or abdominal wall. We performed a systematic review of PubMed and Embase for “External oblique intercostal plane block.” We found five studies and four case reports [3-11]. All the studies and case reports showed successful analgesia with EOIPB. Table 1 shows the current studies and cases in the literature that have used this block for analgesia [3-11 ]. EOIPB is often easily accessible compared to traditional plane blocks in these patients [4,9]. These nerve blocks can offer sustained relief in chronic conditions. The procedure involves identifying the intercostal nerves beneath the external oblique muscle and administering a mixture of local anesthetic and medication to numb the nerve and reduce inflammation. This targeted approach can provide significant and prolonged pain relief for individuals experiencing chronic pain in the chest or abdominal regions. According to the literature review, the potential risks of EOIPB include the risk of pneumothorax from needle insertion and anesthetic toxicity from systemic uptake [12]. If a catheter is used in this block, there is a risk of infection and bleeding at the catheter site [12].

**Table 1:** Current literature on the external oblique intercostal plane block for upper abdominal surgeries

Serial no.	Study	Country	Year of publication	Study design	Number of patients	Indications for external oblique intercoastal plane block	Dosage of anesthetic used
1	Elsharkawy et al. [3]	USA	2021	Retrospective study	22	16 injections, 6 infusions: small bowel resection, partial colectomy, laparoscopic Whipple’s, ileo colectomy, retroperitoneal mass excision, laparoscopic sigmoidectomy, liver transplantation, laparoscopic gastrectomy, laparoscopic proctopexy, exploratory laparotomy, donor nephrectomy, Nissen fundoplication, donor hepatectomy, revision biliary anastomosis, biliary-enteric anastomosis, excision of the ampulla of Vater, renal transplantation, closure enterostomy	For injections: 15 mL of bupivacaine 0.25% was injected bilaterally. For infusions: 10 mL of bupivacaine 0.25% was injected
2	Coşarcan et al. [4]	Turkey	2022	Retrospective study	15	Bariatric surgery	30 mL bupivacaine 0.25% was administered bilaterally
3	Korkusuz et al. [5]	Turkey	2022	Prospective study	40	Laparoscopic cholecystectomy	25 mL of 0.25% bupivacaine bilaterally
4	Doymus et al. [6]	Turkey	2024	Prospective study	30	Laparoscopic sleeve gastrectomy	30 mL bupivacaine 0.25%
5	Kavakli et al. [7]	Turkey	2024	Prospective study	28	Laparoscopic sleeve gastrectomy	20 mL bupivacaine 0.25% bilaterally
6	Coşarcan and Erçelen [8]	Turkey	2022	Case series	3	2 Laparoscopic liver surgery, 1 laparoscopic bariatric surgery	20 mL of subcostal 0.25% bupivacaine bilaterally along with bilateral transversus abdominis plane block or bilateral rectus sheath block
7	White and Ji [9]	USA	2022	Case series	2	Open distal pancreatectomy and emergency cholecystectomy	Patient 1: 20 mL of ropivacaine 0.2% was injected bilaterally into each catheter. A programmed intermittent bolus regimen of ropivacaine 0.2%, 15 mL every 3 h was delivered, and run through each catheter. Patient 2: 40 mL of ropivacaine 0.5% was injected, followed by an intermittent bolus of ropivacaine 0.2%, 20 mL every 2 h
8	O'Donovan and Martin [10]	USA	2021	Case report	1	Laparoscopic cholecystectomy	Bupivacaine 0.1% infusion at 7 cc/h
9	Wilkinson-Maitland et al. [11]	USA	2023	Case report	1	Kasai portoenterostomy in a neonate	Chloroprocaine 1.5%, 2 mL, and post-operative analgesia using 1.5% chloroprocaine 1.3 mL (11 mg/kg/h total) infusion

Since the block is performed superficially, its compressible location is safe for patients on anticoagulation. The superficial location also makes it accessible in obese patients and is easier to perform than neuraxial, transverse abdominis plane block and erector spinae plane blocks. Performing the block and managing it during the procedure does not require patient manipulation and extensive positioning since the patient is placed in the supine position. Since the site of the block is often away from the operative field it does not interfere with the procedure [9,12]. Further, it allows reduction of pain medication and opioid use in the first 24 hours after the procedure compared to other regional plane blocks like TAP block and rectus sheath block [4,6,7,10]. One disadvantage of the block is that lacks visceral analgesic coverage, only provides coverage to the upper abdominal wall, and does not provide analgesia below the umbilicus [3,5].

Further studies are needed to investigate the optimum technique, dosing, volume of analgesic for the block, and scope of the block for various upper abdominal procedures.

## Conclusions

Management of post-surgical neuropathic pain requires a multimodal approach. Interventional techniques, including intercostal plane blocks under ultrasound guidance, offer effective relief when conservative measures fail. Clinicians should consider these options in patients with persistent pain after surgery.
